# Neurovascular coupling dysfunction in encephalopathy: pathophysiological advances and clinical implications

**DOI:** 10.3389/fneur.2025.1522485

**Published:** 2025-05-20

**Authors:** Lvbing Sheng, Xiaoyu Zheng, Zhao Ding, Jianxun Liu, Wenting Song

**Affiliations:** Beijing Key Laboratory of Pharmacology of Chinese Materia Medic, Institute of Basic Medical Sciences of Xiyuan Hospital, China Academy of Chinese Medical Sciences, Beijing, China

**Keywords:** neurovascular unit, neurovascular coupling, endothelin-1, encephalopathy, pathophysiological advances, clinical implications

## Abstract

Neurovascular coupling (NVC) is a sophisticated and vital physiological mechanism that ensures the brain’s intricate balance and optimal performance. It refers to the precise coordination between the brain’s neural activity and the local cerebral blood flow (CBF), which is essential for meeting the metabolic demands of active neurons. This coupling allows for the efficient delivery of oxygen and nutrients to brain regions experiencing increased activity and facilitates the removal of metabolic waste products. In encephalopathy, a collective term for a wide range of conditions that impair brain function, NVC dysfunction has been identified as a key factor contributing to the progression of these disorders and the emergence of clinical symptoms. This comprehensive review aims to explore the complex pathophysiological mechanisms that lead to NVC dysfunction in several encephalopathic conditions. These include but are not limited to Alzheimer’s disease (AD), Parkinson’s disease (PD), cerebral small vessel disease (CSVD), stroke, migraine, traumatic brain injury (TBI) and epilepsy. Across the spectrum of encephalopathies discussed in this review, a unifying molecular target emerges: endothelin-1 (ET-1) and its receptors. ET-1, a potent vasoconstrictor produced by endothelial cells and astrocytes, is intricately linked to NVC dysfunction in these conditions. A thorough understanding of the role of NVC in encephalopathic disorders can inform the development of diagnostic tools and therapeutic strategies. For instance, identifying early markers of NVC dysfunction could facilitate early intervention and potentially slow disease progression. Moreover, targeting the restoration of NVC could become a novel therapeutic approach to mitigate symptoms and improve patient outcomes. This review also proposes new directions for future research, encouraging the exploration of NVC’s complex interactions and its potential as a therapeutic target in the management of encephalopathic conditions.

## 1 Introduction

The brain possesses a highly specialized vasculature that is distinct in both structure and function when compared to vasculature supplying peripheral organs (1). The cerebral vasculature exhibits distinct structural and functional specializations compared to peripheral organs. Its arterial system features robust collateral circulation via arterio-arterial anastomoses, such as the Circle of Willis, which enhances ischemic compensation. Cerebral capillaries are characterized by an exceptionally high pericyte-to-endothelial cell ratio (1:1–1:3), which, alongside tight endothelial junctions and astrocytic endfeet, forms the blood–brain barrier (BBB) to tightly regulate molecular exchange (2, 3). In contrast, peripheral vasculature relies on fenestrated endothelia and looser junctions for permeability. Cerebral veins lack valves, instead draining via intracranial pressure gradients through rigid dural sinuses, while waste clearance is mediated by the glymphatic system, a brain-specific pathway driven by aquaporin-4-dependent cerebrospinal fluid-interstitial fluid exchange (4). Peripheral organs, conversely, depend on conventional lymphatic vessels and valved venous systems for drainage and waste removal. These adaptations collectively ensure cerebral homeostasis, precise metabolic regulation, and protection against ischemic or toxic insults, underscoring the brain vasculature’s unique evolutionary specialization for neuroprotection. To maintain the functional and structural integrity of the brain, it is essential to precisely regulate local blood flow and strategically deliver metabolic substrates to meet the energy demands arising from neuronal activity. Active neurons emit signals that are transmitted to blood vessels, enabling localized modulation of blood flow and ensuring the efficient delivery of bioenergetic substrates. This phenomenon, known as neurovascular coupling, is achieved through a harmonized dialogue between neural and vascular cells (5).

Neurovascular coupling (NVC) is a complex process regulated by both feedforward and feedback mechanisms. Feedforward mechanisms are primarily mediated by nonmetabolic signaling pathways, such as glutamate synaptic signaling, which directly initiate intracellular calcium-dependent cascades that produce vasoactive messengers to increase blood flow (6). Feedback mechanisms involve metabolic factors like oxygen, glucose, and CO2. For example, a drop in oxygen levels or an increase in CO2 can trigger vasodilation to adjust blood flow (6, 7). These two mechanisms work together to maintain the stability of the brain’s internal environment. When feedforward mechanisms function improperly, feedback mechanisms can compensate to ensure appropriate blood flow. This integrated system allows for efficient regulation of CBF in response to neural activity. At the core of NVC function lies the neurovascular unit (NVU) (8), a dynamic assembly encompassing neurons, vascular cells (notably endothelial cells, pericytes, and vascular smooth muscle cells), glial cells (primarily astrocytes, microglia, and oligodendrocytes), and the extracellular matrix. Interactions between neural, glial, and vascular components of the NVU precisely regulate CBF, vascular function, neuroimmune responses, and waste clearance through coordinated intercellular signaling (1). NVC emerges as a fundamental aspect of the NVU, highlighting how neuronal activation can directly generate or transmit signals to glial cells and interneurons, ultimately influencing small blood vessels and regulating blood flow in response to metabolic demands (9). This integrated approach emphasizing the vascular and neural components offers a fresh perspective for comprehensively elucidating the neurovascular mechanisms underlying encephalopathy (10).

From a physiological perspective, neurovascular coupling plays a pivotal role in functional hyperemia, which involves the localized increase in blood flow triggered by neuronal activity. This ensures adequate delivery of oxygen and nutrients to active neurons (11). The intricate tripartite network formed by vessels, neurons, and glial cells orchestrates the dynamic processes underlying the CNS development. Besides the interaction between neurons, glial cells, and endothelial cells, various other cell types and the stromal microenvironment also play crucial roles in shaping and maintaining the neurovascular interface. During the initial stages of CNS vascularization, pericytes are recruited by endothelial cells and contribute to establishing the BBB by decreasing vascular permeability (12). In addition, CNS macrophages regulate vascular fusion via anastomosis. Furthermore, the perivascular basement membrane has a profound impact on CNS development, and defects in pial basal lamina composition can lead to impaired radial glia anchorage and aberrant neuronal migration (12). The neurovascular signaling cascade involves neuronal activation and the release of the neurotransmitter glutamate. This activation stimulates metabotropic glutamate receptors on astrocytic processes, leading to elevated calcium (Ca2+) levels in astrocytes through the activation of inositol trisphosphate receptors. In turn, Ca2 + −dependent release of vasodilatory signals from astrocyte endfeet onto parenchymal arterioles occurs. Astrocytic calcium signaling, particularly via the IP3R2-dependent Gq-GPCR pathway, seems peripheral to neurovascular coupling (13). IP3R2 knockout mice exhibit normal neurovascular responses *in vivo*, with astrocytic calcium increases lagging behind vascular changes and occurring sporadically (14). Genetic activation of the Gq pathway or using sensitive calcium indicators like GCaMP shows no direct effect on baseline or stimulus-induced blood flow (13). Startle-evoked calcium signals in astrocytes follow blood flow changes. Thus, astrocytic calcium signaling may not initiate or sustain neurovascular coupling but could relate to synaptic plasticity or metabolism (15). Neurovascular regulation likely relies more on direct neuronal actions or non-calcium astrocytic mechanisms. The functional role of astrocytes necessitates a comprehensive re-evaluation, with a focus on identifying and characterizing alternative NVC pathways under physiologically relevant conditions. Prominent vasodilatory mediators include vasodilatory arachidonic acid metabolites and K + efflux mediated by activation of large-conductance Ca2 + −activated K + (BK) channels on astrocyte endfeet. This integrated signaling cascade ensures optimal cerebrovascular function and supports neuronal homeostasis in the CNS (11).

**Figure 1:**
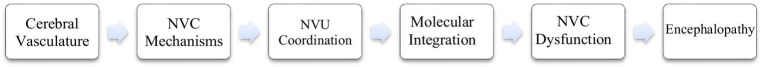
The pivotal role of NVC dysfunction in encephalopathy.

Molecular interactions between the nervous and vascular systems are crucial for maintaining the proper coupling of organ structure and function. Pathways shared by both systems are increasingly acknowledged as key players in facilitating communication between the neuronal compartment and the endothelial cells (16). Both systems share common pathways, emphasizing the overlap and integration of their respective molecular vocabularies. This vocabulary not only regulates vascular and neuronal processes within their respective domains, but also facilitates crosstalk between the neural and vascular systems (17). Furthermore, this common repertoire of molecular signals is critical for facilitating communication between the neural and vascular systems. These findings underscore the intricate and interconnected nature of molecular signaling both within these two vital biological systems and across them. Building on the understanding of molecular interactions between the nervous and vascular systems, it is crucial to recognize that disturbances in NVC can have a substantial effect on brain health. As demonstrated in Figure 1, the pivotal role of NVC dysfunction in encephalopathy is evident.

## 2 Neurovascular coupling dysfunction in encephalopathy

The pathophysiological disruption of NVC serves as a critical mechanism underpinning diverse encephalopathies, including AD, CSVD, stroke, migraine, TBI, and epilepsy. Central to this dysregulation is the aberrant signaling of endothelin-1 (ET-1), a key mediator of NVC impairment. Mechanistically, ET-1 binds to its receptors triggering pathological vasoconstriction, oxidative stress, and neuroinflammatory cascades, which collectively exacerbate cerebral hypoperfusion, metabolic dysregulation, and neuronal injury. Disease-specific manifestations of ET-1 dysregulation include impaired Aβ clearance and endothelial dysfunction in AD, ischemic penumbral expansion and inverse NVC in stroke/subarachnoid hemorrhage, cortical spreading depolarization in migraine, hyperexcitability in epilepsy, and microvascular degeneration with mitochondrial failure in CSVD and TBI. Pharmacological targeting of ET-1 via receptor antagonists (e.g., macitentan) presents a unifying therapeutic strategy to restore neurovascular homeostasis by mitigating vasoconstrictive pathways, enhancing perfusion-metabolic coupling, and counteracting the shared molecular drivers of NVC dysfunction. The onset of encephalopathy is characterized by NVC failure, which disrupts the critical equilibrium between CBF, oxygen delivery, and neuronal metabolic demands. This mismatch impairs cerebral substrate availability—including glucose (essential for ATP synthesis), oxygen (for aerobic metabolism), neurotransmitter precursors (e.g., glutamate), ionoregulatory molecules (Na^+^/K^+^ gradients, Ca^2+^ signaling), and cofactors (thiamine, B12)—culminating in mitochondrial dysfunction, synaptic failure, and neurotoxic metabolite accumulation (e.g., lactate, hyperammonemia). These perturbations drive neuronal energy crisis, excitotoxicity, and oxidative stress, thereby accelerating encephalopathic progression (18). Elucidating the molecular interplay governing physiological and pathological CBF dynamics is thus pivotal for advancing our understanding of cerebral homeostasis and disease pathogenesis (19).

### 2.1 Alzheimer’s disease

The defining neuropathological features of Alzheimer’s disease (AD) comprise extracellular amyloid-*β* (Aβ) plaque deposition and intracellular neurofibrillary tangles (NFTs) composed of hyperphosphorylated tau protein (19). A critical pathogenic component involves multifactorial cerebrovascular dysregulation, characterized by BBB compromise, CBF disturbances, and impaired Aβ vascular clearance mechanisms (20). Aβ oligomers exert direct neurotoxic effects on endothelial integrity and disrupt NVC, potentiating hypoperfusion and metabolic insufficiency. Central to AD pathophysiology is progressive NVC failure, which uncouples activity-dependent neurovascular signaling, thereby depriving neurons of hemodynamic-metabolic support (21, 22). This dysfunction accelerates neurodegenerative cascades through feedforward mechanisms, propagating a self-reinforcing pathogenic cycle of Aβ deposition, tau hyperphosphorylation, and synaptic attrition.

Endothelin-1 (ET-1), a potent vasoconstrictor synthesized by endothelial cells and astrocytes, has emerged as a pivotal mediator of NVC dysfunction in AD (23). Elevated ET-1 levels in AD patients correlate with chronic cerebral hypoperfusion due to sustained microvascular vasoconstriction, which reduces CBF and impairs Aβ clearance mechanisms. Furthermore, ET-1 exacerbates BBB leakage, facilitating the extravasation of neurotoxic plasma proteins and inflammatory mediators into the brain parenchyma (24). This process not only amplifies Aβ deposition and tau hyperphosphorylation but also induces morphological alterations in astrocytic endfeet, such as retraction and edema, alongside diminished expression of glutamate and lactate transporters. Preclinical studies highlight the therapeutic potential of ET-1 receptor antagonists, which attenuate NVC dysfunction, restore CBF, and reduce Aβ and tau pathology in AD models (25). These findings underscore ET-1 as a master regulator linking vascular dysregulation to AD-specific neurodegeneration, offering dual therapeutic avenues to target both vascular and neurodegenerative pathways.

The perturbations in cerebrovascular function in AD are evidenced by NVC dysfunction. Multiple investigations in both rodent and human models of AD have elevated levels of Aβ peptides correlate with diminished resting CBF and attenuated vasodilatory responses within the cerebral vasculature, implicating compromised NVC (26). Pericytes, which are essential for regulating vascular tone and NVC, undergo degeneration in AD, thereby impairing the tripartite signaling network among endothelial cells, astrocytes, and neurons (27). Astrocytes, integral to NVC, detect neuronal signals and release vasodilators such as nitric oxide (NO) to regulate vascular mural cells. However, Aβ-induced endothelial dysfunction disrupts this coordination, leading to attenuated vasodilatory responses and diminished resting CBF (28).

Advancements in neuroimaging have revolutionized the evaluation of NVC dysfunction in AD. Dynamic contrast-enhanced MRI (DCE-MRI) quantifies BBB integrity, while diffusion tensor imaging (DTI-MRI) maps white matter lesions and connectivity. Blood oxygen level-dependent functional MRI (BOLD-fMRI) assesses CBF dysregulation and cerebral metabolic rate of oxygen consumption, whereas T2*-weighted and susceptibility-weighted imaging (SWI-MRI) detect microhemorrhages. Additionally, T1- and T2/FLAIR-weighted imaging quantify regional atrophy. These methodologies offer promising potential for AD diagnosis, facilitating early detection of NVC dysfunction (22, 29). Moreover, dynamic retinal vessel analysis (DVA) constitutes a non-invasive neuroimaging modality that quantifies NVC through high-precision measurement of arteriolar reactivity to flicker light stimuli (30). This functional biomarker platform enables non-contact assessment of microvascular regulatory capacity, with attenuated vasodynamic responses reflecting cerebrovascular autoregulation deficits in aging and neurodegenerative conditions (31). Multidisciplinary applications demonstrate DVA’s sensitivity in detecting preclinical microangiopathy across AD progression (32). Notably, longitudinal studies establish DVA-detected NVC impairment as prodromal biomarker in AD pathogenesis, mediated through neurovascular-metabolic axis dysregulation (30, 31). These advancements hold the potential to enhance our comprehension of NVC dysfunction’s correlation with cognitive decline and neurodegenerative processes in AD, leading to improved treatment strategies and patient outcomes (33).

Longitudinal studies are imperative to elucidate the temporal dynamics of NVC dysfunction and its causal relationship with AD etiology (116). Integrating neuroimaging data with molecular insights into ET-1 signaling and vascular pathology will advance the identification of novel biomarkers and therapeutic targets. ET-1 receptor antagonists, alongside interventions targeting pericytes and astrocyte-endothelial interactions, hold promise for restoring NVC and mitigating AD progression. Furthermore, early detection of NVC impairment through multimodal imaging and retinal assessments may enable timely interventions, improving patient outcomes.

In conclusion, NVC dysfunction represents a critical nexus between vascular dysregulation and neurodegeneration in AD. By unraveling the roles of ET-1, pericytes, and astrocytes, and leveraging cutting-edge neuroimaging tools, the field is poised to develop precision therapies that address both the vascular and neurodegenerative dimensions of this devastating disease.

### 2.2 Parkinson’s disease

Parkinson’s disease (PD), a progressive neurodegenerative disorder characterized by dopaminergic neuron loss in the substantia nigra pars compacta (SNc) and intraneuronal protein aggregation, is associated with profound NVC dysfunction that exacerbates disease progression (34, 35). Abnormal neuronal activity played a primary role in neurovascular dysfunction with relatively preserved perfusion. This suggests that neuronal impairment may precede and be more severe than reduced perfusion in early stages of PD (36). Through the analysis of CBF and low-frequency fluctuation amplitude (ALFF) in PD patients, researchers identified NVC deficits primarily in the motor circuits of the striato-thalamo-cortical region and motor control areas (36). Simultaneously, both dopaminergic and nondopaminergic systems contribute to neurovascular changes, with the right dorsolateral superior frontal gyrus identified as a potential target for treatment in PD patients (37).

The pathophysiological rationale for targeting ET-1 in PD-associated NVC dysfunction arises from its dual roles in cerebral vasoconstriction and neuroinflammation. Intranigral ET-1 induces focal ischemia via vascular smooth muscle ETA receptor activation, reducing blood flow, causing tissue necrosis, and driving nigrostriatal dopaminergic degeneration (38). Additionally, ET-1 modulates microglial inflammation via ETB receptors, promoting pro-inflammatory cytokine release that exacerbates neuronal damage. Neural stem cell (NSC) transplantation demonstrates therapeutic potential by differentiating into functional neurons and astrocytes that integrate into host circuitry, restore synaptic connections, and facilitate vascular normalization (39). This body of work identifies ET-1 as a pivotal therapeutic target in Parkinson’s disease PD-associated neurovascular dysfunction, while highlighting NSC-based interventions as promising strategies for achieving comprehensive repair through neuroprotection, angiogenic modulation, and synaptic network reintegration.

Clinically, while dopamine precursor therapy (e.g., L-DOPA) remains a mainstay for motor symptoms, it frequently induces dyskinesia (LID) associated with aberrant NVC. L-DOPA alters regional cerebral blood flow (rCBF) and metabolism in basal ganglia output nuclei, where high-density D1 dopamine receptors modulate interactions between endothelial cells, astrocytes, and microvasculature structure (40, 41). The resultant rCBF-metabolism mismatch in the basal ganglia-thalamocortical circuit highlights NVC regulation as a critical target for LID management (42). Emerging strategies, such as deep brain stimulation (DBS) (43, 44), show promise in restoring neurovascular connectivity, particularly in advanced PD patients unresponsive to conventional therapies, by normalizing CBF and neuronal activity. These findings collectively emphasize the complex interplay between neurovascular dysfunction and PD pathogenesis, advocating for targeted interventions—including ET-1 receptor antagonism, NVC-regulating neuromodulation, and regenerative strategies—to address both neuronal and vascular components of the disease.

### 2.3 Cerebral small vessel disease

Cerebral small vessel disease (CSVD) represents a spectrum of vascular pathologies affecting intracranial microvessels, leading to heterogeneous clinical manifestations and neuroimaging markers, including white matter hyperintensities (WMH), lacunar infarcts, cerebral microbleeds (CMBs), and cortical atrophy (16, 45). Central to its pathogenesis is NVC dysfunction, a critical mechanism underlying the imbalance between neuronal metabolic demand and CBF regulation. This disruption precipitates chronic hypoperfusion, impaired clearance of neurotoxic metabolites, and progressive white matter injury, ultimately contributing to cognitive decline and dementia.

NVC dysfunction in CSVD leads to chronic hypoperfusion, impaired metabolic support, and accelerated white matter injury. ET-1 exacerbates this dysfunction by inducing sustained microvascular constriction, which reduces CBF and compromises the clearance of neurotoxic metabolites such as Aβ (46). Elevated levels of ET-1 in CSVD patients are correlated with BBB disruption, which is characterized by perivascular protein leakage and the infiltration of inflammatory mediators into the brain parenchyma. These processes contribute to the development of white matter hyperintensities (WMH) and lacunar infarcts (47). Preclinical studies demonstrate that ET-1 receptor antagonists attenuate neurovascular uncoupling, restore CBF autoregulation, and mitigate white matter damage in CSVD models, suggesting ET-1 signaling directly links vascular dysregulation to neurodegeneration (48). The current investigation identifies ET-1 as a linchpin in the NVC dysfunction of CSVD, delineating a therapeutic strategy that targets ET-1 receptor signaling to simultaneously ameliorate vascular dysfunction and suppress maladaptive neuroinflammatory cascades.

Interactions among the components of the NVU are recognized as vital for maintaining homeostasis within the brain’s internal environment. Structural and functional disruptions in diverse NVU elements, encompassing molecular damage, organelle dysfunction, and cellular injury, which give rise to reduced microvascular perfusion and compromised clearance pathways, contribute to the vulnerability of the NVU to CSVD (45). These disruptions impair microvascular perfusion and metabolite clearance, perpetuating a vicious cycle of ischemia, hypoxia, and neuronal injury. A meta-analyses has demonstrated that progressive NVC dysfunction exhibits a temporal correlation with the advancement of CSVD markers, particularly WMH volume expansion and CMB accumulation, thereby underscoring its dual significance as both a diagnostic biomarker and an interventional target (47). Advanced neuroimaging techniques, including functional near-infrared spectroscopy (fNIRS) and arterial spin labeling (ASL), have elucidated the correlation between NVC impairment and CSVD severity. Resting-state functional MRI (rs-fMRI) reveals aberrant NVC patterns in regions such as the left superior frontal gyrus, which may serve as a biomarker for memory deficits (9). Emerging multimodal approaches integrating fNIRS, diffuse correlation spectroscopy (DCS), and 3D pseudo-continuous ASL (3D-pASL) offer comprehensive insights into neuronal excitability, vascular reactivity, and oxygen metabolism (49). Such technologies not only enhance pathophysiological understanding but also provide frameworks for monitoring therapeutic interventions. A research project integrating rs-fMRI and 3D-pASL is designed to characterize patterns of NVC dysfunction in patients with CSVD, while investigating the underlying mechanisms contributing to CSVD-associated cognitive impairment (9).

Based on the above, NVC dysfunction lies at the nexus of CSVD pathophysiology, bridging microvascular pathology to cognitive impairment. Elucidating ET-1-driven mechanisms and leveraging advanced imaging biomarkers are critical for developing precision therapies. Future research should prioritize translational studies validating NVC restoration as a therapeutic paradigm, thereby addressing both vascular and neurodegenerative components of CSVD in a unified strategy.

### 2.4 Stroke

Stroke is defined as an impairment in cerebral perfusion, thereby inducing the necrosis of neural tissue in specific cerebral regions. It can be primarily subcategorized into two major types: ischemic stroke, which ensues as a consequence of vascular occlusion leading to tissue death; and hemorrhagic stroke, which occurs due to the rupture of a blood vessel (10). Post-stroke astrogliosis is particularly pronounced in astrocytes endfeet around cerebral blood vessels, serving as a critical biomarker for the underlying pathophysiological changes that transpire post-stroke (42). This evidence suggests that the reductions in CBF and impairments in NVC observed after a stroke may be associated with dysfunctions within astrocytic populations.

#### 2.4.1 Subarachnoid hemorrhage

Subarachnoid hemorrhage (SAH) is a rare but severe subtype of hemorrhagic stroke, accounting for 10% of all strokes. It refers to bleeding within the area between the arachnoid membrane and the pia mater surrounding the brain (50, 51). The main causes of SAH are typically the rupture of an aneurysm or an arteriovenous malformation (AVM). SAH is characterized by pathophysiological features such as the initial bleeding event, neuroinflammation, cerebral vasospasm, and delayed cerebral ischemia (DCI), which can result in severe neurological complications. Increasing evidence indicates that a dysregulated reversal of NVC could be a pivotal factor in the pathophysiology of SAH, manifesting in the propagation of spreading depolarization waves and within the NVU in response to localized neuronal activation (52).

The presence of subarachnoid blood significantly disrupts communication within the NVU, leading to an abnormal NVC. In over 90% of cortical brain slices from SAH model animals, a significant increase in astrocytic endfoot Ca2 + levels was observed, leading to vasoconstriction instead of the expected dilation response during neuronal activity (53, 117). This abnormality in NVC resulted in a decrease in oxygen and nutrient flow to active neurons, further exacerbating the pathological conditions in the brain. To study this phenomenon, researchers utilized multiphoton confocal imaging and infrared differential interference contrast (IR-DIC) microscopy to observe the effects of SAH on neurovascular coupling in cortical brain slices from SAH model rats. Analysis of the data revealed that heightened spontaneous Ca2 + events post-SAH boosted BK channel activity, which in turn increased basal K + levels in the perivascular space, ultimately causing a disruption in NVC (54). These findings highlight a crucial alteration in the intricate relationship between neurons and blood vessels following SAH, shedding light on potential therapeutic targets for intervention.

The presence of blood in the subarachnoid space elicits an inflammatory response and modifies vascular reactivity. That is an abnormal shift towards vasoconstriction referred to inverse NVC. This maladaptive response can result in diminished CBF and exacerbate ischemic conditions in the affected cerebral regions. It not only engenders immediate neurological deficits subsequent to SAH but may also evoke secondary injury machineries that progressively deteriorate the integrity of brain tissue (55). Notwithstanding the inversion of vascular response polarity subsequent to SAH, both neurally modulated dilation and constriction during SAH encompass comparable mechanistic constituents: augmented intracellular Ca2 + concentrations and K + efflux via endfoot large-conductance potassium BK channels (56). By discerning the underpinning mechanisms of inverse NVC and its contribution to post-SAH pathophysiology, clinicians can more proficiently execute targeted therapeutic regimens aimed at reestablishing normal hemodynamic functionality and enhancing patient prognoses following such critical incidents.

ET-1 has emerged as a central therapeutic target in NVC dysfunction following SAH due to its upregulated expression post-SAH. This upregulation positively correlates with prolonged mean transit time (MTT) and contributes to cerebral microcirculatory impairment via potent vasoconstriction, increased vascular resistance, and regional perfusion deficits, ultimately exacerbating delayed ischemic injury (57, 58). ET-1 induces vasoconstriction via activation of endothelin receptor type A (ETAR), which is overexpressed in SAH. This overexpression results in reduced CBF and impaired NVC. Silencing ETAR ameliorates cerebrovascular hemodynamic disturbances and alleviates neurological impairments by inhibiting ERK/KLF4-dependent phenotypic modulation in smooth muscle cells (SMCs), underscoring its dual function as both a mediator of microvascular injury and a therapeutic strategy for neurovascular repair through targeted suppression of maladaptive SMC remodeling (59). Future research should focus on elucidating the intricate interplay between ET-1, ETAR, and NVC dysfunction, as well as exploring novel therapeutic approaches to mitigate the detrimental effects of SAH on neurovascular integrity.

#### 2.4.2 Ischemic stroke

Ischemic stroke (IS) accounts for approximately 80% of all strokes and is a prevalent cerebrovascular disorder that occurs when a blood vessel in the brain is suddenly blocked, cutting off blood flow to the affected regions (50). It is essential for adequate blood flow to be restored to the brain after IS, which relies not only on open cerebral arteries and collaterals but also on compensatory mechanisms in the brain’s microcirculation. Research into the NVU has also led to new treatment approaches for this condition (60, 61). When brain activity increases, blood vessels in the brain respond by dilating, which leads to an increase in CBF. This understanding is key to developing effective strategies for managing IS (62).

The cerebral blood flow-fractional amplitude of low-frequency fluctuations (CBF-fALFF) metric represents a multimodal biomarker integrating hemodynamic perfusion (CBF, quantified through arterial spin labeling MRI) and neurophysiological oscillatory activity (fALFF, derived from resting-state fMRI BOLD signal oscillations within 0.01–0.08 Hz) (63). It correlates regional oxygen delivery with spontaneous neuronal synchronization. Disruptions in CBF-fALFF coherence are linked to impaired NVC in neurodegenerative and cerebrovascular disorders. fMRI studies reveal decreased CBF-fALFF coupling in chronic ischemic stroke patients with basal ganglia involvement (64). Moreover, local deviations in the CBF-fALFF ratio suggest potential functional reconfiguration contralateral to the lesion. In acute ischemic stroke models, elevated 20-HETE synthesis near the infarct zone attenuates capillary NVC (63). Laser speckle contrast imaging (LSCI) shows that optogenetic stimulation elicits weaker hemodynamic responses in ischemic tissue, with regional CBF correlating to distance from the ischemic core (65). Excitatory neuron activation in non-lesioned regions enhances NVC recovery, unlike in the peri-infarct area (66). These outcomes highlight the potential of targeting intact penumbral regions for excitatory stimulation to restore perfusion post-stroke.

ET-1 is a key player in NVC dysfunction during ischemic stroke. It contributes to cerebrovascular dysfunction through JNK MAPK-dependent signaling pathways and excessive NMDA receptor activation. Local administration of ET-1 leads to prolonged cortical hypoperfusion, with a 30–50% decrease from baseline that lasts up to 16 h. This is accompanied by delayed hyperperfusion and progressive vascular remodeling, which mirrors the dynamics observed in human stroke (67, 68). Pathologically, ET-1 intensifies BBB disruption through aquaporin-4 upregulation and exacerbates neuroinflammation. Persistent astrogliosis, microglial activation, and widespread necrotic neuronal loss extending beyond the ischemic core regions are observed (68, 69). Pharmacological blockade of ET receptors (ETRA and ETRB) reduces edema volume by 50% and enhances sensorimotor outcomes by normalizing vascular reactivity and inhibiting pro-inflammatory mediators (69). Mechanistic investigations reveal that tPA-mediated activation of NMDA receptors following stroke induces JNK phosphorylation, which raises ET-1 levels and impairs autoregulation. These effects can be reversed by NMDA-sparing tPA variants, such as tPA-A296–299, which restore the balance of the cAMP/p38-JNK pathway while maintaining fibrinolytic efficacy (70). The research underscores the pivotal role of ET-1 as a central hub connecting excitotoxicity, hemodynamic failure, and inflammatory cascades in cerebral ischemia. It advocates for targeted receptor antagonism or the application of modified thrombolytics as effective approaches to reestablish neurovascular homeostasis.

The NVC concept plays a crucial role in identifying potential biomarkers associated with stroke and understanding the pathophysiological processes at different stages of the disease. By combining insights from morphological studies (71–73) that examine the distribution and levels of specific biomarkers like aquaporin-4 (AQP4), amino acids, and metabolic byproducts with results from neuroradiological assessments that assess disruptions in CBF, brain edema progression, and the ischemic penumbra dynamics, we can improve cerebral stroke diagnosis. The intricate connection between the pathophysiological events and the neurovascular repair mechanisms in IS correlates with the homeostatic state of the NVU (74). Consequently, further investigation into NVU-targeted therapeutics could potentially broaden the therapeutic options for IS and deepen our understanding of the pathophysiological mechanisms underlying NVC dysfunction in the context of IS.

### 2.5 Migraine

Migraine is a type of primary headache disorder with a pathophysiological mechanism involving trigeminal activation leading to a vascular response. The pain from migraines is typically felt in various regions of the head, including the frontal, temporal, parietal, occipital, and higher cervical areas. A significant aspect related to migraines is Cortical Spreading Depolarization (CSD), a condition characterized by waves of neuronal activity silence after depolarization, often associated with migraine aura (75). Elevated plasma ET-1 levels during migraine attacks suggest its role in mediating vasoconstriction. In experimental models, ET-1 induces strong cerebral vasoconstriction mediated primarily through ETA receptors, which can be blocked by selective antagonists. However, studies using ET-1 receptor antagonists in spreading depression (SD), a phenomenon linked to migraine aura, show no effect on the oligemic phase of SD (76). The evidence suggests that although ET-1 might play a role in vasoconstriction during migraine, it is probably not a key factor in the blood flow alterations associated with SD. This implies that ET-1 antagonists may have restricted therapeutic efficacy for migraine treatment.

Various studies (77, 78), including imaging investigations and animal research, have revealed complex changes in cortical neurovascular connections during migraine aura and CSD episodes. Animal studies (79, 80) suggest that CSD could trigger sensitization mechanisms in both peripheral and central regions, contributing to migraine headaches. Additionally, certain medications utilized for migraine prevention have demonstrated efficacy in reducing susceptibility to cortical spreading depression (CSD) in animal models. These include CGRP inhibitors (81) (e.g., Erenumab, Fremanezumab, Galcanezumab), which target the calcitonin gene-related peptide pathway; anticonvulsants (82) (e.g., Divalproex sodium, Topiramate), which stabilize neuronal membranes; calcium channel blockers (83) (e.g., Verapamil), which inhibit calcium influx; and other agents such as Amitriptyline and Gabapentin. These pharmacological agents operate through diverse mechanisms, including receptor blockade, ion channel modulation, and neurotransmitter regulation. This multifaceted approach offers various avenues for advancing treatment strategies aimed at migraine prevention. It is evident that cortical neurovascular disruptions coincide with migraine aura symptoms, with CSD standing out as a plausible explanation for these occurrences (84). However, further exploration is required to fully understand the relationship between cortical nerve and vascular components during migraine aura episodes. This research could provide valuable insights into the underlying mechanisms of migraines and potentially lead to more targeted treatment strategies.

To determine the vein density from Susceptibility-Weighted Imaging (SWI)-MRI, in subjects with migraine and healthy controls; and to assess whether it relates to Resting-State functional MRI (RS-fMRI). Results point towards an increase in vein density in subjects with migraine, when compared to healthy controls (85). In addition, the association between GM vein density and ALFF found in healthy subjects was lost in migraine. These results support the idea of abnormalities in the neurovascular coupling in migraine (86). Numerous studies (77, 87, 88) utilizing single-photon emission computed tomography (SPECT) or the Xenon-133 blood flow technique during interictal phases have identified abnormalities in CBF among migraine patients, predominantly characterized by hypoperfusion across various cortical regions or asymmetrical distributions of cortical blood flow. Besides, Multi-modal MRI is a new method to assess the impact of chronic pain to the brain which could be used to detect NVC dysfunction in chronic migraine patients (89).

### 2.6 Traumatic brain injury

Traumatic brain injury (TBI) represents a complex interplay of primary and secondary injury cascades. The primary injury arises from direct biomechanical forces, resulting in immediate structural damage to neurons, glia, and vasculature, including contusions, hematomas, and axonal shearing. In contrast, secondary injury evolves over hours to days post-trauma, driven by molecular and cellular responses that amplify neural damage (90). This delayed phase offers critical therapeutic opportunities, particularly in addressing NVC dysfunction—a hallmark of TBI pathophysiology linked to cerebral hypoperfusion and metabolic derangements (91). The primary injury associated with TBI is irreversible, prompting an emphasis on preventive measures designed to reduce neuroinflammation triggered by immune cells and cytokines/chemokines. Whereas secondary injuries can be more harmful than the initial injuries, they offer potential avenues for therapeutic intervention (92).

Central to NVC impairment is the pathological upregulation of ET-1, a potent vasoconstrictor synthesized by endothelial cells, macrophages, and hippocampal neurons following TBI. Elevated ET-1 levels correlate temporally with disrupted cerebrovascular autoregulation and sustained reductions in regional blood flow, exacerbating secondary neuronal injury (93). Mechanistically, endothelin receptor A (ETrA) exhibits preferential upregulation in vascular smooth muscle and cortical/hippocampal neurons post-TBI, driving vasoconstrictive dominance, while endothelin receptor B (ETrB) remains confined to endothelial cells with limited compensatory roles (94). This imbalance is further modulated by NMDA receptor activation and JNK-mediated signaling pathways. Notably, wild-type tissue plasminogen activator (tPA) exacerbates ET-1 overexpression, whereas the tPA-A^296–299^ variant attenuates ET-1 synthesis by preserving cAMP levels, thereby restoring autoregulatory vasodilation and mitigating hippocampal necrosis (70).

Concomitant with ET-1 dysregulation, TBI-induced BBB disruption triggers NVU dysfunction, altering inflammatory mediator release and angiogenic responses. In repetitive mild TBI (rmTBI) models, NVC deficits manifest as reduced red blood cell velocity, microvessel constriction, and impaired leukocyte-endothelial interactions (95). Neuronal-specific AAV-mediated overexpression of protein arginine methyltransferase 7 (PRMT7) has emerged as a promising intervention, restoring NVC, curtailing BBB leakage, and enhancing mitochondrial respiration, thereby highlighting PRMT7’s neuroprotective potential (96).

CSD is a common pathological electrophysiological process that occurs after TBI, as evidenced by both animal and human studies (97–99). The occurrence of CSD during TBI can result in a mismatch between the oxygen consumption needs of mitochondria and the supply provided by the vasculature, leading to NVC disturbances and mitochondrial dysfunction (100). CSD is associated with heightened mitochondrial reactive oxygen species (ROS), astrocytic damage, and pericyte dysfunction, perpetuating secondary injury and metabolic crisis (101). These disturbances underscore the need for targeted therapies to mitigate CSD-driven NVC impairment.

Taken together, TBI-induced NVC dysfunction arises from multifaceted interactions between ET-1 dysregulation, NVU disruption, and CSD-mediated metabolic stress. Therapeutic strategies focusing on ET-1 modulation (e.g., tPA variants), PRMT7 overexpression, and CSD inhibition hold promise for restoring cerebrovascular homeostasis and improving clinical outcomes. Future research must prioritize translational approaches to bridge these pathophysiological insights into effective clinical interventions.

### 2.7 Epilepsy

Epilepsy is a chronic neurological disorder characterized by the manifestation of recurrent, spontaneous, and synchronized seizures. The pathophysiological mechanisms underlying epilepsy, known as epileptogenesis, encompass a complex interplay of alterations within neurons, neuroglia, and the endothelium. These modifications lead to both structural and functional deficits within the NVU, ultimately culminating in the onset of spontaneous epilepsy (102). During epileptic seizures, synchronized excessive neuronal discharges result in a pronounced elevation of the oxygen metabolic rate, placing supra-normal demands on the brain’s autoregulatory mechanisms. As a consequence, normal NVC may be significantly compromised (103, 104).

The evolution of hemodynamic responses to seizures over time suggests a gradual disconnect between neurovascular functions at the capillary and arteriolar levels, ruling out hypoxia as an immediate factor in microvascular dysfunction and cellular injury (105). In laboratory settings, it has been observed that diminishing capillary responsiveness occurs despite sufficient oxygen and consistent enhancement of respiration during seizures (106). A widely used 4-aminopyridine (4-AP) seizure model reliably triggers focal seizures with appropriate intervals between episodes, providing a valuable platform for studying the dynamics of neurovascular events in specific seizure foci (107). In epilepsy, neuronal networks generate epileptiform discharges that lead to modifications in astrocytic and CBF dynamics.

Emerging evidence highlights dynamic alterations in NVC during seizures (106, 108). Stereoelectroencephalography studies reveal spatio-temporal hemodynamic-electrophysiological coupling patterns, with initial hyperperfusion at seizure onset zones transitioning to hypo-perfusion during propagation, correlating with electrophysiological evolution stages (109, 110). These biphasic perfusion dynamics suggest neurovascular decoupling mechanisms that may influence seizure termination and epileptogenic progression. This aligns with hypotheses that NVC dysfunction mediates epileptogenesis via BBB disruption and metabolic dysregulation. Such findings underscore the NVU’s role as both a biomarker source and therapeutic target for precision epilepsy interventions.

ET-1 emerges as a pivotal mediator in neurovascular dysfunction during epileptogenesis, with dual receptor-dependent mechanisms underpinning its pathophysiological role. Activation of ETA receptors induces focal ischemia through sustained cerebral hypoperfusion and hypoxia, triggering excitotoxic neuronal injury via glutamate/GABA imbalance. This is evidenced by prolonged seizures and hippocampal lesions in immature rats (111, 112). In contrast, ETB receptor activation drives non-ischemic seizures through inflammatory pathways, characterized by elevated leukotriene B4/prostaglandin E2 ratios, independent of vascular compromise (113). Critically, the severity of chronic epilepsy correlates with the extent of ET-1-induced hippocampal damage, highlighting a feedforward loop between neuronal injury and hyperexcitability. These receptor-specific pathways—ETA-mediated ischemia-excitation coupling and ETB-dependent neuroinflammation—collectively disrupt neurovascular homeostasis, positioning ET-1 as a dual therapeutic target for mitigating both vascular and inflammatory components of epileptogenesis (112, 113).

The limited efficacy of antiepileptic drugs and prevalence of pharmacoresistance necessitate deeper investigation into epileptogenesis mechanisms, particularly NVU dysfunction involving endothelial cells, astrocytes, and pericytes (114, 115). Understanding these mechanisms and targeting ET-1 pathways may offer new therapeutic strategies to address the vascular and inflammatory aspects of epilepsy, potentially improving outcomes for patients with this debilitating disorder. Future research should focus on elucidating the intricate interactions between neurovascular components and exploring novel therapeutic interventions that target these pathways to mitigate epileptogenesis and its associated comorbidities.

## 3 Conclusions and future directions

In the context of brain pathologies, NVC dysfunction is increasingly recognized as pivotal to the underlying pathophysiological mechanisms. NVC entails the precise matching of local blood flow with neuronal metabolic demand, a process often derailed in encephalopathy. The impairment of NVC can be attributed to several factors, such as the presence of vascular risk factors that induce endothelial dysfunction, leading to a diminished capacity for vasodilation in response to neuronal activity. Additionally, neuroinflammatory processes can result in the release of cytokines and other mediators that further compromise the integrity of the blood–brain barrier and vascular reactivity. The ensuing dysregulation in the cerebral microenvironment can lead to reduced clearance of neurotoxic metabolites, culminating in a feedforward loop that exacerbates neuronal injury and disease progression. The intricate relationship between these mechanisms underscores the multifactorial nature of NVC dysfunction and its critical role in the pathogenesis of encephalopathy. Targeting ET-1 signaling represents a cross-cutting therapeutic strategy to restore NVC and BBB homeostasis in encephalopathies. By addressing this shared pathway, interventions could mitigate neurovascular uncoupling, reduce metabolic stress, and slow disease progression, offering a unified approach to diverse neurological disorders. Despite considerable evidence indicated that the NVC dysfunction are closely related to many encephalopathy, some underlying mechanisms remain largely unknown. Accordingly, understanding NVC dysfunction in encephalopathy has important clinical implications.

The clinical significance of investigating NVC dysfunction in brain pathologies lies in its potential to unravel early pathological changes that precede overt clinical symptoms. NVC dysfunction can serve as sensitive indicators of the onset and progression of neurological disorders, offering a unique window into the brain’s metabolic and hemodynamic responses. As an early diagnostic tool, impaired NVC may facilitate the identification of at-risk individuals before significant neuronal loss occurs, allowing for timely intervention. Moreover, the assessment of NVC can inform therapeutic strategies by providing a dynamic measure of the cerebrovascular health and its capacity to adapt to metabolic demands. Additionally, monitoring NVC can be instrumental in evaluating the efficacy of treatments aimed at preserving or restoring vascular reactivity and cognitive function. The ability to non-invasively measure NVC may also contribute to the development of personalized medicine approaches, tailoring interventions to an individual’s unique vascular and neuronal status. A variety of non-invasive and in-vivo techniques are currently used for assessing NVC in the diagnosis and management of neurological disorders. Advanced neuroimaging modalities such as dynamic contrast-enhanced magnetic resonance imaging (DCE-MRI), diffusion tensor imaging (DTI-MRI), and blood oxygen level-dependent functional MRI (BOLD-fMRI) are widely utilized. DCE-MRI assesses blood–brain barrier integrity, DTI-MRI examines white matter connectivity, and BOLD-fMRI investigates hemodynamic responses. Additionally, dynamic retinal vessel analysis (DVA) measures retinal vessel diameter changes in response to flicker light stimulation, providing insights into microvascular function. Other techniques include transcranial Doppler (TCD) ultrasound for assessing cerebral blood flow velocity, optical coherence tomography (OCT) for visualizing retinal and optic nerve structures, and laser speckle contrast imaging (LSCI) for mapping hemodynamic responses. These methods enable clinicians and researchers to evaluate NVC dysfunction, monitor disease progression, and assess the efficacy of therapeutic interventions in various neurological conditions. Overall, the exploration of NVC in encephalopathy underscores its importance as a prognostic, diagnostic, and therapeutically relevant parameter in the clinical management of neurological conditions.

Current research on NVC has made significant strides in understanding the temporal and regional associations between neural activity and CBF responses. However, a crucial limitation lies in overlooking major structural factors that play a role in NVC, such as the anatomical structure of cerebral vasculature and organ structure regulating CBF (3). Moving forward, it is imperative for future research to integrate structural neuroimaging features alongside functional neuroimaging to gain a comprehensive understanding of NVC. While current studies heavily rely on non-physiological ex vivo preparations and non-human models under sedation, there is a need to shift towards *in vivo* human models to bridge the gap between animal studies and human application. Another critical challenge in NVC research is the limited exploration of primary risk factors and the utilization of small sample sizes in clinical studies. To address this issue, a more streamlined analysis using standardized software is essential. This approach will allow for the incorporation of larger datasets to predict risk factors associated with NVC impairment across different populations and age groups. Looking ahead, future research holds promise in developing a clinically relevant cerebrovascular outcome measure to evaluate therapeutic interventions for various risk factors and clinical conditions. The widespread adoption of standardized software will not only propel the field of NVC forward but also deepen our understanding of this complex mechanism. Ultimately, integrating structural features with functional assessments and standardized analyses will pave the way for more effective management strategies in neurological disorders.

A comprehensive elucidation of the NVC’s role in encephalopathic disorders is pivotal for advancing the development of diagnostic acumen and therapeutic interventions. The identification of early biomarkers indicative of NVC impairment could serve as a critical juncture for initiating preemptive medical interventions. The strategic targeting of NVC restoration presents a promising frontier in therapeutic innovation. Restoration of NVC requires targeted pharmacological modulation of endothelial, neuronal, and glial pathways. Key therapeutic strategies include: (1) Endothelial nitric oxide synthase (eNOS) potentiation via cilostazol or statins to enhance vasodilatory NO bioavailability, countering age- or disease-related endothelial dysfunction; (2) Anti-inflammatory agents (e.g., TNF-*α* inhibitors, COX-2 inhibitors) to mitigate neuroinflammation-induced astrocytic endothelin-1 overproduction, which disrupts capillary perfusion; (3) Vasoactive neuropeptide modulation, such as pituitary adenylate cyclase-activating polypeptide (PACAP) analogs, to restore astrocyte-mediated vascular tone regulation; (4) Metabolic reprogramming via SIRT1 activators (e.g., resveratrol derivatives) to improve mitochondrial energetics in endothelial cells and neurons; and (5) Endothelin receptor antagonists (e.g., macitentan) to reverse pathological vasoconstriction in cerebral small vessel disease. Preclinical studies demonstrate that combinatorial approaches synergistically improve CBF-neuronal activity matching, with ongoing clinical trials evaluating their translational potential in early Alzheimer’s disease and diabetic encephalopathy. The approach could ameliorate the symptomatology associated with encephalopathic disorders and enhance the overall prognosis for patients. By normalizing the compromised NVC, it may be possible to restore the intricate balance between cerebral blood flow and neuronal activity, which is often disrupted in these pathological states. Future studies will need to analyze neurovascular interactions in a temporal and area-specific manner to elucidate the intrinsic mechanisms that regulate cellular phenotype and its integrated functionality. The integration of advanced imaging techniques with single-cell transcriptomics and proteomics is expected to yield spatiotemporal maps clarifying interactions at the neurovascular interface, enhancing our understanding of the development and maintenance of the NVU. Neurovascular research is set to expand the molecular framework related to key mechanisms underlying neuronal and vascular communication, facilitating a better comprehension of complex developmental processes and the continuous homeostatic regulation of the NVU (7). Ongoing research in neurovascular biology is poised to expand the molecular repertoire that underlies the complex mechanisms of neuronal-vascular crosstalk. This expansion will pave the way for innovative strategies in the prophylaxis, diagnostic precision, and therapeutic management of conditions intricately linked with NVC. As the field progresses, it is expected to uncover novel molecular targets and pathways that could be harnessed for the mitigation of diseases that have their pathological roots in NVC dysfunction.

In summary, the study of NVC dysfunction in encephalopathy is a rapidly evolving field that offers promising insights into disease mechanisms and potential therapeutic strategies. As mounting researches continue to elucidate the physiopathologic mechanism of NVC, it expectantly holds the key to unlocking new treatments and improving the lives of patients with encephalopathy.
